# Market competition in banking and asset-liability maturity mismatch of non-financial firms: Evidence from Vietnam

**DOI:** 10.1371/journal.pone.0344896

**Published:** 2026-07-06

**Authors:** Thi Minh Hue Phan, Japan Huynh

**Affiliations:** Faculty of Finance and Banking, Ho Chi Minh City Open University, Ho Chi Minh City, Vietnam; Liverpool John Moores University, UNITED KINGDOM OF GREAT BRITAIN AND NORTHERN IRELAND

## Abstract

This study examines the effect of bank competition on corporate investment-financing maturity mismatch, utilizing a panel dataset of 498 listed firms in Vietnam from 2008 to 2024. Bank competition is measured using both structural and non-structural indicators, allowing for a nuanced assessment of market dynamics. The findings reveal a robust positive association between bank competition and maturity mismatch, suggesting that intensified competition leads firms to increase their reliance on short-term debt relative to long-term investment needs. This relationship holds under multiple robustness checks, including alternative variable constructions, fixed effects specifications, crisis period exclusions, and instrumental variable approaches. Mechanism analyses indicate that bank competition affects firms’ debt maturity structures, increasing both the proportion and scale of short-term borrowing. Heterogeneity tests further show that this effect is stronger among firms with higher bank debt dependence, greater financial constraints, and higher borrowing costs, while it is weaker in capital-intensive sectors.

## 1. Introduction

Firms’ maturity structure, which reflects how well the maturities of their assets and liabilities are aligned, is a fundamental component of liquidity management and financial stability. In theory, long-term investments should be financed with long-term debt to avoid refinancing risks, especially under credit market uncertainty [1]. However, in practice, many firms finance long-term assets with short-term liabilities, creating maturity mismatches. This issue is particularly pronounced in emerging markets, where limited access to long-term credit exacerbates the problem [2]. Such mismatches can heighten refinancing risks, trigger bankruptcy, destabilize financial systems, and hinder economic development [3,4]. A growing body of literature has investigated the drivers of maturity mismatch from both firm-level and macroeconomic perspectives, highlighting the roles of corporate characteristics, institutional factors, and broader economic conditions. Despite this progress, the role of banking market structure, particularly bank competition, remains underexplored. This is an important omission, as bank competition can shape firms’ access to credit, financing costs, and borrowing structures.

Theoretically, the effect of bank competition on maturity mismatch is ambiguous. On the one hand, the market power hypothesis suggests that stronger competition facilitates long-term financing and reduces mismatch [5,6]. On the other hand, the information hypothesis argues that intensified competition discourages relationship lending and promotes short-term transactional credit, which can increase maturity mismatch [7,8]. These competing views underscore the need for empirical analysis to clarify how bank competition affects corporate maturity mismatch.

To fill the gap in the existing literature, this paper empirically investigates how bank competition affects corporate investment-financing maturity mismatch, using a novel dataset from Vietnam. We compile a comprehensive panel of 498 listed firms over the 2008–2024 period. Bank competition is assessed through both structural measures (including the concentration ratio of the largest banks and the Herfindahl-Hirschman index) and a non-structural measure – the Lerner index, which captures banks’ pricing power relative to marginal cost. This multidimensional approach is motivated by the recognition that different competition indicators reflect distinct facets of market dynamics and are not interchangeable [9]. While structural measures emphasize market share concentration, the non-structural Lerner index captures conduct-based pricing behavior, allowing us to explore how varying dimensions of competitive pressure influence firms’ reliance on short-term debt to fund long-term investments.

For the empirical analysis, we employ the dynamic system generalized method of moments (GMM) estimation to address the potential persistence in maturity mismatch and mitigate endogeneity concerns. We further perform a range of robustness checks to ensure the reliability of our findings. These include the use of alternative measures for both bank competition and maturity mismatch, adjustments in fixed effects structures, the exclusion of periods of financial and public health crises, and instrumental variable estimations. Building upon the baseline analysis, we conduct mechanism tests to examine how bank competition could reshape firms’ debt maturity structure, thereby influencing maturity mismatch. In addition, we explore heterogeneity across different types of firms and industries, including those with varying degrees of bank debt exposure, financial constraints, borrowing costs, and industry capital intensity. These additional analyses enrich our understanding of the conditions under which bank competition may aggravate or alleviate corporate investment-financing misalignment.

Vietnam presents a compelling case for examining the influence of bank competition on firms’ investment-financing maturity structures, owing to several distinctive institutional and financial characteristics. The country’s financial system is predominantly reliant on banks, as capital markets, particularly the bond market, remain relatively underdeveloped. Consequently, bank loans constitute the principal source of external financing for most enterprises [10], which magnifies the role of banking sector conditions in shaping corporate financial strategies. Additionally, Vietnam has implemented wide-ranging reforms in its banking industry since joining the World Trade Organization in 2007. These reforms have included the privatization of state-controlled banks, the entrance of foreign competitors, and the consolidation of financially weaker institutions—developments that have significantly altered the competitive landscape [11]. Moreover, Vietnamese firms tend to be smaller in scale and face more severe financial constraints compared to their counterparts in advanced or peer emerging economies. This makes them particularly responsive to variations in credit conditions [12]. Furthermore, the prevalence of poor financial disclosure and limited enforcement of corporate governance exacerbates information asymmetries, which can influence banks’ willingness to offer long-term credit and firms’ decisions regarding debt maturity [13,14].

This paper makes two main contributions to the literature. First, it extends the literature on bank competition and corporate finance by examining its effect on investment-financing maturity mismatch, thereby integrating investment and financing decisions within a unified framework. In doing so, it employs both structural and non-structural measures of bank competition to more comprehensively capture market dynamics. Second, the paper advances the understanding of how bank competition shapes corporate financing behavior by identifying the underlying mechanisms and sources of heterogeneity. In particular, it highlights the role of debt maturity structure and firm-specific characteristics in shaping the relationship between bank competition and maturity mismatch.

## 2. Literature review and research hypothesis

This section develops the theoretical foundation for examining the effect of bank competition on corporate investment-financing maturity mismatch. It is organized into three parts. First, we review the literature on the determinants of corporate maturity mismatch and the role of bank competition to identify the remaining research gap. Second, we discuss the market power view, which predicts that bank competition may reduce maturity mismatch by improving firms’ access to long-term credit. Third, we examine the information hypothesis, which suggests that bank competition may increase maturity mismatch by weakening relationship lending and encouraging short-term financing. These competing perspectives motivate our empirical investigation.

### 2.1. Determinants of corporate maturity mismatch and the role of bank competition

Corporate investment-financing maturity mismatch arises when firms finance long-term investments with short-term liabilities, exposing them to refinancing risks and financial instability. Prior literature has identified a broad set of determinants shaping firms’ maturity structures. At the firm level, maturity mismatch is influenced by internal characteristics such as performance dynamics, corporate governance, ownership structures, and the role of large shareholders [15–19]. At the macro level, institutional and economic conditions, including central bank communication, capital account liberalization, public enforcement, geopolitical uncertainty, and climate change, also play an important role in shaping firms’ financing structures [20–23].

While this literature highlights the multifaceted nature of maturity mismatch, it pays limited attention to the role of banking market structure, particularly bank competition. This omission is important because banks are the primary providers of external finance in bank-based financial systems, where they influence not only the availability and cost of credit but also its maturity structure. A substantial body of research shows that bank competition affects corporate financial behavior along several dimensions, including investment decisions, external financing costs, financial asset allocation, and access to credit, especially for financially constrained firms [24–29].

However, existing studies predominantly focus on the quantity and pricing of credit rather than its maturity structure. As a result, the implications of bank competition for the alignment between financing maturities and investment horizons remain largely unexplored. This gap suggests that bank competition may be an important but underexamined determinant of corporate investment-financing maturity mismatch, motivating the need for a more direct empirical investigation.

### 2.2. Bank competition and maturity mismatch: the market power view

The key theoretical foundation is the market power hypothesis, which argues that heightened bank competition improves firms’ access to credit by reducing the monopolistic power of lenders [5,30]. In highly concentrated banking sectors, banks often restrict access to long-term financing or provide such loans under stringent conditions. In contrast, greater competition incentivizes banks to allocate credit more efficiently and expand their offerings of long-term loan products to attract and retain clients [9,31]. As a result, firms gain improved access to credit instruments that are better aligned with the duration of their capital investment needs, thereby reducing maturity mismatch.

In addition, stronger competition typically leads to lower borrowing costs, particularly for long-term debt [6]. Declining interest rates and lending fees reduce the financial burden of long-term borrowing, making it more attractive for firms to align their liabilities with investment horizons. Banks also differentiate themselves by offering customized long-term financing products that support extended investment cycles [21]. Collectively, these dynamics contribute to more stable capital structures, allowing firms to reduce their reliance on short-term funding for long-term assets. Consequently, easier and more affordable access to long-term credit lowers the likelihood that firms may finance fixed investments with short-term liabilities, thereby mitigating maturity mismatch.

These theoretical insights are supported by recent empirical evidence. Li et al. [32] show that bank competition is negatively associated with the probability of investment-financing maturity mismatch among SMEs in China. Their findings reinforce the view that competitive banking environments enhance firms’ ability to align financing structures with investment needs.

Based on the above arguments, we propose the following hypothesis:

Hypothesis A. Bank competition tends to reduce corporate investment-financing maturity mismatch.

### 2.3. Bank competition and maturity mismatch: the information hypothesis

In contrast to arguments suggesting that bank competition reduces investment-financing maturity mismatch, an opposing body of theory highlights mechanisms through which heightened competition may exacerbate mismatch. This perspective is grounded in the information hypothesis, which posits that intensified competition diminishes banks’ incentives and capacity to maintain long-term lending relationships. As banks compete more aggressively for market share, they increasingly adopt transactional lending practices that prioritize speed and flexibility over in-depth borrower monitoring and relationship building [7,33]. Consequently, banks become less inclined to extend long-term credit, particularly in cases where evaluating project risk requires firm-specific information [8,34]. The reduced availability of long-term debt compels firms to rely more heavily on short-term borrowing, irrespective of the long-term nature of their investment projects.

Firms operating in such credit environments respond rationally to the prevailing lending conditions. Short-term loans, which are often more affordable and accessible due to competitive pricing, are favored for their lower costs and expedited approval processes [35]. Even firms engaged in long-term investments may opt for short-term financing, rationalizing the mismatch by assuming continued access to credit rollovers. This behavior is further reinforced by overconfidence in refinancing prospects, as intense bank competition can create the perception of uninterrupted credit availability [36]. Thus, bank competition not only alters the supply-side structure of credit markets but also influences firms’ liability management decisions, thereby increasing the likelihood of maturity mismatch.

Accordingly, we propose the competing hypothesis:

Hypothesis B. Bank competition tends to increase corporate investment-financing maturity mismatch.

## 3. Research design

### 3.1. Variables

#### 3.1.1. Measurement for bank competition.

Accurately capturing the degree of bank competition is essential for understanding how financial market dynamics shape corporate behavior, particularly in terms of investment-financing decisions. However, measuring competition is inherently challenging, as no single indicator can fully encapsulate its complexity [9]. Structural indicators offer insights into market concentration but often fail to reflect actual competitive behavior or pricing power. Conversely, non-structural measures are more aligned with market conduct but may not account for institutional scale or distribution. Recognizing these limitations, this study adopts a comprehensive approach that combines both structural and non-structural measures of bank competition. This framework enables a more nuanced analysis and helps prevent potential misinterpretation of results that could arise from relying on a single metric.

The first measure employed is the three-bank concentration ratio (CR), which is defined as the cumulative market share of the three largest banks in terms of total assets. This measure reflects the dominance of the largest institutions within the banking system. Higher values of CR indicate greater banking market concentration, which is typically interpreted as a lower level of competition.

The second measure is based on the Herfindahl-Hirschman index, calculated as the sum of the squared market shares (based on total assets) of all banks operating in the system (HHI). Unlike CR, which focuses on the top segment of the market, HHI captures the entire market structure. As with CR, larger HHI values signify higher concentration and hence lower competitive intensity.

The third measure used to capture bank competition is the Lerner index, a non-structural indicator that reflects the pricing power of individual banks. This index measures the extent to which a bank can set prices above marginal costs, with higher values indicating greater market power and thus lower levels of competition. Specifically, for each bank *i* in year *t*, the Lerner index is calculated as:


$$\begin{array}{c} {Lerner}_{it\;}=\frac{P_{it\;}\;-\;{MC}_{it\;}}{P_{it\;}} \end{array}$$ (1)

where $P_{it\;}$ represents the price of bank outputs, calculated as the ratio of total revenue (including interest and non-interest income) to total assets. The term ${MC}_{it\;}$ refers to the marginal cost of producing banking services, which is not directly observable and therefore estimated through a cost function. The estimation involves three input prices: labor, physical capital, and funding. The full specification of the translog cost function and the marginal cost derivation procedures are provided in the study of Berger et al. [37], which provides a comprehensive methodological framework for this approach.

To obtain a national-level indicator, we compute the asset-weighted average of the Lerner indices across all banks in each year. This aggregation accounts for the relative size of banks and ensures that larger institutions exert proportionately greater influence on the annual index. The Lerner index, therefore, captures variation in market power and can effectively track shifts in competitive intensity over time.

#### 3.1.2. Measurement for corporate maturity match.

To assess the extent of mismatch between investment needs and financing maturity, we adopt a methodology that is widely accepted in the literature on corporate finance and maturity structure [15, 16, 22, 38]. Our approach follows the logic that firms should ideally finance long-term investments using long-term capital sources, such as internal cash flows, equity, and long-term debt. When these long-term sources are insufficient, and firms rely instead on short-term borrowing to fund long-term investments, a maturity mismatch arises.

We begin by computing a financing gap. This gap is calculated as capital expenditure minus the sum of internal funds and external long-term funds. Specifically, internal funds are defined as the sum of net cash flow from operating activities and proceeds from the disposal of fixed assets. External long-term funds include the increase in long-term debt and net equity financing. Thus, the financing gap is calculated as: *[capital expenditure – (net cash flow from operating activities + disposal of fixed assets + increase in long-term debt + net proceeds from equity financing)].*

The increase in long-term debt is measured as the change in long-term debt from the previous year to the current year, adjusted by adding non-current liabilities that are due within one year. Once the gap is obtained, we divide it by total assets to eliminate firm size effects. The resulting ratio is our continuous measure of maturity mismatch, denoted as IFMM. A higher IFMM value indicates a more severe maturity mismatch, suggesting that a greater share of long-term investment is likely funded by short-term liabilities.

### 3.2. Empirical model

The baseline empirical model is designed to evaluate the impact of bank competition on corporate investment-financing maturity mismatch as follows:


$$\begin{array}{c} {IFMM}_{i,t}\;={\;\alpha }_{\text{0}}\;+{\;\alpha_{\text{1}}\times IFMM}_{i,t-\text{1}}\;+\;\alpha_{\text{2}}\times {Competition}_{t-\text{1}}\;+\;\beta \times {Firm}_{i,t-\text{1}}\;+\;\gamma \times {Country}_{t-\text{1}}\;+\;\alpha_{i}\;+\;\varepsilon_{i,t\;} \end{array}$$ (2)

The core components of the model include the maturity mismatch as the dependent variable (IFMM), the bank competition indicator (Competition) as the main explanatory variable (using CR, HHI, or Lerner separately), and a comprehensive set of control variables that account for firm-specific (Firm) and macroeconomic characteristics (Country). These controls, detailed in Table 1, include firm-level indicators such as firm size, leverage, profitability, state ownership, liquidity, asset tangibility, and market valuation (Tobin’s Q). In addition, country-level controls such as the growth rate of the money supply and stock market returns are included to account for broader relevant time-varying influences. We also include dummy variables for the global financial crisis (2008–2009) and the COVID-19 pandemic (2020–2021) to capture systemic macroeconomic shocks. These control variables are well-grounded in the existing empirical literature on the determinants of investment-financing maturity alignment [15,16,22,39]. All explanatory variables, including the bank competition indicators and control variables, are lagged by one period in the regressions. This lag structure serves two purposes. First, it helps mitigate potential endogeneity concerns by reducing the simultaneity between current financing-investment decisions and contemporaneous competition measures. Second, it reflects the realistic assumption that firms respond to financial and economic conditions with a delay.

**Table 1:** Definitions of variables and summary statistics.

	Mean	SD	10th	Median	90th	Calculations
IFMM	−0.138	0.252	−0.405	−0.065	0.038	Difference between the maturity of firm assets and financing, as detailed in subsection 3.1.2
Size	27.177	1.572	25.280	27.049	29.212	Natural logarithm of total assets
Leverage	0.469	0.224	0.154	0.472	0.763	Ratio of total liabilities to total assets
StateOwn	0.228	0.253	0.000	0.111	0.558	Percentage of total shares held by the state
ROA	0.068	0.072	0.004	0.051	0.157	Net income divided by total assets
CashRatio	0.101	0.103	0.009	0.066	0.239	Cash and cash equivalents divided by total assets
PPE	0.196	0.183	0.016	0.136	0.463	Ratio of fixed tangible assets (PPE) to total assets
TobinQ	1.396	1.223	0.420	1.010	2.800	Market value of equity plus total debt, divided by total assets
CR	0.391	0.030	0.360	0.384	0.418	Combined market share of the three largest banks, based on total assets
HHI	0.077	0.009	0.069	0.075	0.081	Sum of squared market shares of all banks in the market (based on total assets)
Lerner	0.276	0.109	0.164	0.261	0.476	A non-structural measure calculated as (price – marginal cost)/price, aggregated using asset weights of banks
MoneySupply	0.163	0.060	0.081	0.162	0.262	Annual growth rate of the M2 money supply
StockReturn	0.079	0.277	−0.327	0.121	0.480	Annual growth rate of the VN-Index

The model includes firm fixed effects ($\alpha_{i}$) to control for unobserved heterogeneity across firms that remains constant over time. However, time fixed effects are excluded from the estimation. Including year dummies would absorb all year-specific variation, thereby eliminating the identifying variation in our key explanatory variables, particularly the bank competition indicators, which vary at the year level.

The analysis employs the dynamic panel model, estimated using the two-step system GMM. This estimation technique is appropriate given the dynamic nature of corporate financing-investment decisions and the likely persistence of maturity mismatch over time [40,41]. Moreover, the system GMM estimator effectively handles potential endogeneity that may arise from reverse causality, omitted variable bias, or measurement errors [42,43]. The model uses internal instruments constructed from lagged levels and differences of the regressors to ensure the consistency and unbiasedness of the estimates. To validate the GMM estimations, we report the results of some standard diagnostic tests. The Hansen test of overidentifying restrictions is used to assess the validity of the instruments, ensuring that they are not correlated with the error term. Additionally, the Arellano-Bond test for second-order serial correlation (AR(2)) in the differenced residuals is conducted to confirm the absence of higher-order autocorrelation.

### 3.3. Data

Our analysis of the relationship between bank competition and corporate investment-financing maturity mismatch in Vietnam is supported by two primary data sources: firm-level and bank-level financial statements. We obtain data on publicly listed firms from the Ho Chi Minh Stock Exchange (HOSE) and the Hanoi Stock Exchange (HNX). Financial information, including balance sheet items, cash flow statements, and income indicators, is retrieved from the FiinPro database, a well-established and comprehensive source of financial data in Vietnam. Following standard practices in the corporate finance literature, we exclude firms in the financial and utility sectors due to their distinct regulatory environments and operational characteristics, which may introduce bias into the analysis of typical corporate investment-financing behavior. Firms with insufficient data required to construct the key variables are excluded from the sample. The final sample for empirical analysis comprises 498 non-financial firms, contributing 6,878 firm-year observations spanning the period from 2008 to 2024.

Bank competition measures are constructed using detailed financial and operational data from 38 commercial banks, resulting in 610 bank-year observations. Bank-level data are also sourced from the FiinPro database. In addition, macroeconomic variables, such as the growth rate of the money supply and stock market performance, are obtained from the same source. To mitigate the influence of extreme outliers, all continuous firm-level variables used in the regressions are winsorized at the 1st and 99th percentiles.

Table 1 presents the summary statistics for all key variables used in the analysis. The reported distributions highlight substantial variation across variables, particularly with regard to corporate maturity mismatch and bank competition. The average value of the maturity mismatch variable (IFMM) is −0.138, with a standard deviation of 0.252. The 10th percentile stands at −0.405, while the 90th percentile is 0.038. These figures suggest a relatively pronounced tendency among Vietnamese firms to fund long-term investments using short-term financing, reflecting the prevalence of maturity mismatch in the sample. The wide dispersion in IFMM values further indicates significant heterogeneity in financing behavior across firms, which supports the empirical relevance of exploring its determinants through regression analysis. The descriptive statistics for bank competition measures also show meaningful variation across the sample period, implying structural shifts in Vietnam’s banking landscape. These patterns are consistent with prior findings reported by Huynh [44], enhancing the credibility and representativeness of our sample. In addition, a correlation matrix (not reported here for brevity) is constructed to test for potential multicollinearity among explanatory variables. The results confirm that severe multicollinearity is not present, supporting the validity of the multivariate regression framework used in our analysis.

## 4. Main analyses

### 4.1. Baseline estimations

The estimation results in Table 2 are based on the system GMM approach, and the validity of this estimation method is supported by standard diagnostic tests. The Hansen test indicates that the instruments used in the model are appropriate and not correlated with the error term, and the Arellano-Bond test for second-order autocorrelation confirms that there is no evidence of serial correlation in the differenced residuals. Further, the coefficient on the lagged dependent variable is statistically significant across all model specifications, confirming the persistence of maturity mismatch over time. These diagnostic results are also consistent across all GMM regressions reported in the subsequent tables, providing strong statistical support for the reliability of the dynamic GMM specifications.

**Table 2:** Bank competition and maturity mismatches: baseline results.

	(1) IFMM	(2) IFMM	(3) IFMM
Lagged DV	0.181***	0.179***	0.179***
	(0.017)	(0.014)	(0.018)
CR	−0.575**		
	(0.292)		
HHI		−3.000***	
		(0.468)	
Lerner			−0.219***
			(0.080)
Size	0.018***	0.014***	0.015***
	(0.003)	(0.002)	(0.003)
Leverage	0.010	0.028	0.021
	(0.020)	(0.017)	(0.020)
StateOwn	0.053***	0.053***	0.052***
	(0.012)	(0.011)	(0.012)
ROA	−0.139**	−0.142***	−0.118**
	(0.059)	(0.052)	(0.060)
CashRatio	0.197***	0.212***	0.207***
	(0.039)	(0.034)	(0.039)
PPE	−0.026	−0.005	−0.021
	(0.020)	(0.017)	(0.020)
TobinQ	−0.031***	−0.031***	−0.030***
	(0.004)	(0.003)	(0.004)
Crisis	−0.008	−0.029***	−0.052***
	(0.025)	(0.011)	(0.011)
Covid	−0.002	−0.001	−0.004
	(0.007)	(0.005)	(0.007)
MoneySupply	−0.087	−0.190***	−0.002
	(0.063)	(0.048)	(0.078)
StockReturn	−0.001	0.013	−0.001
	(0.012)	(0.009)	(0.011)
Firm FE	Yes	Yes	Yes
Observations	6,380	6,380	6,380
Number of firms	498	498	498
Number of instruments	125	125	125
AR(1) test	0.000	0.000	0.000
AR(2) test	0.710	0.739	0.736
Hansen test	0.165	0.252	0.196

The dependent variable in all specifications is investment-financing maturity mismatch (IFMM). Significance levels: * p < 0.1; ** p < 0.05; *** p < 0.01. The numbers in parentheses are standard errors clustered at the firm level.

In our empirical approach, the three bank competition measures (CR, HHI, and Lerner) are each used separately in the regressions. This allows us to evaluate the consistency of results across different dimensions of competition, thereby strengthening the robustness of the findings. The results reported in columns (1) and (2) of Table 2 correspond to CR and HHI, which are structural measures of bank competition. Both coefficients are negative and statistically significant, indicating that greater banking concentration, which implies lower competitive pressure, is associated with a reduction in maturity mismatch. This suggests that firms in more concentrated banking environments tend to align their investment and financing maturities more closely, relying less on short-term debt for long-term investment needs. Column (3) reports the results for Lerner, a non-structural indicator that captures pricing behavior. Similar to the structural measures, the coefficient on Lerner is also negative and significant. This reinforces the finding that lower competition, as reflected in the greater pricing power of banks, is linked to improved maturity alignment at the firm level. In other words, firms operating in less competitive banking environments tend to finance long-term investments more prudently, relying less on short-term debt.

The consistent relationship observed across all three specifications provides strong empirical support for Hypothesis B of the paper, which posits that heightened bank competition may induce firms to increase their reliance on short-term financing for long-term investments, thereby exacerbating maturity mismatch. Our findings contrast with those of Li et al. [32], who document that bank competition reduces the likelihood of investment-financing maturity mismatch among SMEs in China. As bank competition intensifies, their results suggest that SMEs are more likely to obtain longer-term loans, thereby minimizing mismatch risks. Several important distinctions between the two studies may explain this difference. Li et al. [32] rely on financial capacity survey data, focus exclusively on SMEs, and capture bank competition through bank branch density. In contrast, our analysis relies on audited financial statements of listed firms and employs a range of structural and non-structural competition measures based on financial indicators at the bank level. These variations in data sources, firm characteristics, and competition proxies may shape how bank competition influences corporate financing behavior. Recognizing these differences, we further conduct mechanism analyses to clarify the underlying channels behind our findings and to reinforce the credibility and interpretability of our empirical results.

### 4.2. Robustness checks

#### 4.2.1. Alternative measures of bank competition.

As the first part of the robustness analysis, we construct alternative versions of our bank competition measures to evaluate the sensitivity of the findings. First, for the structural indicators, we substitute total loans for total assets when computing market shares. This adjustment yields two alternative measures: the loan-based concentration ratio (CR_loan) and the loan-based Herfindahl-Hirschman index (HHI_loan). These proxies reflect credit market dominance rather than balance sheet size, offering a different lens through which market concentration is assessed. Second, we refine the computation of the Lerner index by excluding the cost of funds from the translog cost function. This follows the approach of Ariss [45] and aims to reduce potential upward bias in the Lerner index that may arise from incorporating deposit pricing distortions. The modified Lerner index is then aggregated across banks using a simple arithmetic average, rather than an asset-weighted mean, to generate the annual value for the overall banking market.

Beyond these three modified measures, we also introduce the Boone index [46], which captures competition through the relationship between firm performance and cost efficiency. The intuition behind this measure is that in more competitive markets, marginal cost efficiency is more strongly rewarded. Formally, the Boone index is estimated from the following log-linear regression model:


$$\begin{array}{c} ln(pi)\;=\;\alpha \;+\;\beta {\times ln(MC}_{i}) \end{array}$$ (3)

where *p*_*i*_ denotes the profits of bank, *MC*_*i*_ represents the estimated marginal cost, and *β* is the Boone coefficient. A more negative value of *β* indicates a higher level of competition, as it implies that less efficient banks are penalized more severely in terms of profitability. Hence, a larger (i.e., less negative) Boone index implies lower competition. The index is computed annually across all banks to capture year-to-year variation in competitive pressure. For further robustness, we estimate the Boone index using pooled ordinary least squares (Boone1), and also apply a system GMM estimator (Boone2), following the methodology of Schaeck and Cihák [47].

We substitute our primary competition variables with these revised measures in all baseline regressions. The resulting estimates, reported in Table 3, are consistent with the baseline analysis, confirming that our core findings regarding the impact of bank competition on maturity mismatch are robust to alternative definitions of competitive intensity.

**Table 3:** Robustness tests: alternative measures of bank competition.

	(1) IFMM	(2) IFMM	(3) IFMM	(4) IFMM	(5) IFMM
Lagged DV	0.185***	0.184***	0.185***	0.186***	0.187***
	(0.014)	(0.014)	(0.014)	(0.014)	(0.014)
CR_loan	−0.384***				
	(0.127)				
HHI_loan		−1.877***			
		(0.474)			
Lerner_average			−0.088***		
			(0.014)		
Boone1				−1.223***	
				(0.325)	
Boone2					−0.919***
					(0.271)
Size	0.013***	0.012***	0.013***	0.014***	0.015***
	(0.002)	(0.002)	(0.002)	(0.002)	(0.002)
Leverage	0.035**	0.034*	0.034*	0.026	0.020
	(0.018)	(0.018)	(0.018)	(0.017)	(0.017)
StateOwn	0.046***	0.045***	0.047***	0.044***	0.048***
	(0.011)	(0.011)	(0.011)	(0.011)	(0.011)
ROA	−0.106**	−0.114**	−0.116**	−0.134***	−0.140***
	(0.053)	(0.052)	(0.052)	(0.052)	(0.052)
CashRatio	0.214***	0.211***	0.215***	0.201***	0.206***
	(0.035)	(0.034)	(0.035)	(0.034)	(0.034)
PPE	0.001	0.001	−0.003	−0.004	−0.006
	(0.017)	(0.017)	(0.017)	(0.017)	(0.017)
TobinQ	−0.033***	−0.032***	−0.032***	−0.031***	−0.031***
	(0.003)	(0.003)	(0.003)	(0.003)	(0.003)
Crisis	−0.046***	−0.029**	−0.060***	−0.066***	−0.063***
	(0.010)	(0.012)	(0.010)	(0.010)	(0.010)
Covid	0.002	0.003	0.009*	0.010**	0.007
	(0.005)	(0.005)	(0.005)	(0.005)	(0.005)
MoneySupply	−0.038	0.004	−0.055	−0.043	−0.086*
	(0.052)	(0.055)	(0.048)	(0.051)	(0.047)
StockReturn	0.009	−0.001	0.011	0.023***	0.031***
	(0.009)	(0.009)	(0.009)	(0.009)	(0.010)
Firm FE	Yes	Yes	Yes	Yes	Yes
Observations	6,380	6,380	6,380	6,380	6,380
Number of firms	498	498	498	498	498
Number of instruments	125	125	125	125	125
AR(1) test	0.000	0.000	0.000	0.000	0.000
AR(2) test	0.672	0.662	0.694	0.742	0.718
Hansen test	0.167	0.135	0.259	0.300	0.595

The dependent variable in all specifications is investment-financing maturity mismatch (IFMM). Significance levels: * p < 0.1; ** p < 0.05; *** p < 0.01. The numbers in parentheses are standard errors clustered at the firm level.

#### 4.2.2. Alternative measures of maturity mismatches.

As an additional robustness check, we construct two alternative measures of corporate maturity mismatch to validate the reliability of our primary IFMM variable [48]. The first proxy, denoted as IFMM1, is defined as *[(short-term debt/total debt) – (short-term assets/total assets)].* This measure captures the net reliance on short-term debt after accounting for short-term assets available for liquidity coverage. The second proxy, IFMM2, is defined as *[(long-term assets – long-term liabilities – equity)/long-term assets]*. This indicator reflects the portion of long-term investments not supported by long-term capital sources, thus highlighting structural maturity misalignment.

We replace our primary maturity mismatch variable with two alternative proxies, and the results, presented in Table 4, continue to support our original findings. While some regressions exhibit changes in significance levels, the overall consistency of the results highlights the robustness of the observed relationship between bank competition and maturity mismatch.

**Table 4:** Robustness tests: alternative measures of maturity mismatches.

	(1) IFMM1	(2) IFMM1	(3) IFMM1	(4) IFMM2	(5) IFMM2	(6) IFMM2
Lagged DV	0.585***	0.582***	0.585***	0.799***	0.798***	0.774***
	(0.020)	(0.020)	(0.019)	(0.017)	(0.017)	(0.018)
CR	−0.295**			−0.786**		
	(0.121)			(0.317)		
HHI		−1.013**			−2.382*	
		(0.449)			(1.278)	
Lerner			−0.005			−0.481***
			(0.030)			(0.102)
Size	−0.007***	−0.007***	−0.006***	0.057***	0.059***	0.071***
	(0.002)	(0.002)	(0.002)	(0.008)	(0.008)	(0.009)
Leverage	−0.037**	−0.039**	−0.041**	0.012	0.027	−0.022
	(0.017)	(0.017)	(0.016)	(0.060)	(0.057)	(0.062)
StateOwn	−0.016	−0.016	−0.012	−0.128***	−0.131***	−0.133***
	(0.011)	(0.011)	(0.011)	(0.044)	(0.044)	(0.044)
ROA	0.065	0.063	0.083*	0.329**	0.400***	0.274*
	(0.046)	(0.047)	(0.044)	(0.164)	(0.154)	(0.165)
CashRatio	−0.014	−0.015	−0.026	0.181	0.167	0.003
	(0.038)	(0.038)	(0.036)	(0.156)	(0.156)	(0.161)
PPE	0.017	0.017	0.015	0.298***	0.294***	0.338***
	(0.017)	(0.017)	(0.016)	(0.068)	(0.068)	(0.069)
TobinQ	−0.002	−0.002	−0.002	−0.040***	−0.041***	−0.040***
	(0.002)	(0.002)	(0.002)	(0.009)	(0.009)	(0.009)
Crisis	−0.015	−0.011	−0.036***	0.004	0.004	−0.019
	(0.011)	(0.013)	(0.007)	(0.023)	(0.025)	(0.021)
Covid	−0.022***	−0.020***	−0.014***	−0.118***	−0.113***	−0.042**
	(0.006)	(0.005)	(0.005)	(0.018)	(0.017)	(0.021)
MoneySupply	−0.150***	−0.161***	−0.161***	0.332***	0.342***	0.109
	(0.040)	(0.039)	(0.044)	(0.124)	(0.124)	(0.129)
StockReturn	0.008	0.006	0.011	−0.041*	−0.047**	−0.025
	(0.008)	(0.008)	(0.007)	(0.021)	(0.021)	(0.021)
Firm FE	Yes	Yes	Yes	Yes	Yes	Yes
Observations	5,086	5,086	5,086	6,369	6,369	6,369
Number of firms	469	469	469	498	498	498
Number of instruments	126	126	126	125	125	125
AR(1) test	0.000	0.000	0.000	0.000	0.000	0.000
AR(2) test	0.197	0.197	0.194	0.701	0.701	0.694
Hansen test	0.103	0.104	0.164	0.215	0.189	0.115

The dependent variables are investment-financing maturity mismatch (IFMM1 and IFMM2). Significance levels: * p < 0.1; ** p < 0.05; *** p < 0.01. The numbers in parentheses are standard errors clustered at the firm level.

#### 4.2.3. Alternative fixed effects.

To further test the robustness of our empirical results, we substitute the firm fixed effects used in the baseline model with industry fixed effects. This adjustment allows us to control for unobserved heterogeneity at the industry level rather than at the individual firm level, thereby capturing common structural characteristics, regulatory environments, or competitive dynamics that may influence firms’ investment-financing behavior within specific sectors. By doing so, we can examine whether the observed relationship between bank competition and maturity mismatch holds when firm-specific time-invariant effects are replaced with broader industry-level controls.

In Table 5, the estimation results under this alternative fixed-effect specification remain consistent with those of the baseline model. The coefficients on all three bank competition measures retain their negative signs and statistical significance, reaffirming the relationship between bank competition and investment-financing maturity mismatch. These findings reinforce the robustness of our conclusions and suggest that the core results are not driven by either firm- or industry-level fixed effects.

**Table 5:** Robustness tests: alternative fixed effects.

	(1) IFMM	(2) IFMM	(3) IFMM
Lagged DV	0.181***	0.193***	0.179***
	(0.014)	(0.013)	(0.013)
CR	−0.315**		
	(0.140)		
HHI		−1.254**	
		(0.495)	
Lerner			−0.129***
			(0.045)
Size	0.003	0.008	0.013*
	(0.006)	(0.005)	(0.007)
Leverage	−0.073**	−0.063**	−0.074*
	(0.036)	(0.031)	(0.040)
StateOwn	−0.040	−0.019	0.014
	(0.031)	(0.024)	(0.031)
ROA	−0.162**	−0.126**	0.171**
	(0.066)	(0.061)	(0.087)
CashRatio	0.108**	0.126***	0.371**
	(0.046)	(0.042)	(0.169)
PPE	−0.006	−0.009	0.079
	(0.037)	(0.031)	(0.107)
TobinQ	−0.028***	−0.030***	−0.067***
	(0.005)	(0.004)	(0.010)
Crisis	−0.035**	−0.023	−0.054***
	(0.015)	(0.017)	(0.011)
Covid	−0.011*	−0.005	0.019***
	(0.006)	(0.005)	(0.006)
MoneySupply	−0.175***	−0.166***	−0.196***
	(0.054)	(0.049)	(0.060)
StockReturn	0.019**	0.018**	0.027***
	(0.009)	(0.008)	(0.010)
Industry FE	Yes	Yes	Yes
Observations	6,380	6,380	6,380
Number of firms	498	498	498
Number of instruments	125	125	125
AR(1) test	0.000	0.000	0.000
AR(2) test	0.770	0.644	0.844
Hansen test	0.103	0.311	0.327

The dependent variable in all specifications is investment-financing maturity mismatch (IFMM). Significance levels: * p < 0.1; ** p < 0.05; *** p < 0.01. The numbers in parentheses are standard errors clustered at the firm level.

#### 4.2.4. Estimations without the crisis periods.

For another robustness check, we re-estimate our baseline regressions after excluding the years associated with major economic disruptions, specifically the global financial crisis and the COVID-19 pandemic. These periods may introduce structural breaks or exceptional policy interventions that could distort normal banking operations and corporate behavior. By removing these years, we aim to ensure that our findings are not driven by crisis-related anomalies or short-term shocks. Although this adjustment reduces both the time span of the panel and the number of firm-year observations, the results remain consistent with those from the full-sample analysis. As displayed in Table 6, the coefficients on the bank competition measures continue to exhibit a negative and statistically significant relationship with maturity mismatch, further confirming the robustness and general applicability of our findings across more stable economic periods.

**Table 6:** Robustness tests: dropping the crisis periods.

	(1) IFMM	(2) IFMM	(3) IFMM
Lagged DV	0.179***	0.183***	0.177***
	(0.019)	(0.019)	(0.019)
CR	−0.689***		
	(0.202)		
HHI		−3.757***	
		(0.948)	
Lerner			−0.158***
			(0.039)
Size	0.013***	0.014***	0.018***
	(0.003)	(0.003)	(0.003)
Leverage	0.044**	0.037*	0.007
	(0.022)	(0.022)	(0.023)
StateOwn	0.037***	0.041***	0.040***
	(0.013)	(0.013)	(0.013)
ROA	−0.144**	−0.147**	−0.199***
	(0.072)	(0.072)	(0.073)
CashRatio	0.255***	0.262***	0.223***
	(0.042)	(0.042)	(0.043)
PPE	−0.008	−0.015	−0.017
	(0.023)	(0.024)	(0.024)
TobinQ	−0.036***	−0.036***	−0.038***
	(0.005)	(0.005)	(0.004)
MoneySupply	−0.202***	−0.254***	−0.233***
	(0.066)	(0.071)	(0.067)
StockReturn	0.013	0.019	−0.002
	(0.015)	(0.015)	(0.013)
Firm FE	Yes	Yes	Yes
Observations	4,252	4,252	4,252
Number of firms	487	487	487
Number of instruments	72	72	72
AR(1) test	0.000	0.000	0.000
AR(2) test	0.398	0.373	0.376
Hansen test	0.153	0.144	0.107

The dependent variable in all specifications is investment-financing maturity mismatch (IFMM). Significance levels: * p < 0.1; ** p < 0.05; *** p < 0.01. The numbers in parentheses are standard errors clustered at the firm level.

#### 4.2.5. Estimations with external instrumental variables.

Our estimations thus far have incorporated several strategies to address potential endogeneity concerns. In detail, we use one-period lagged explanatory variables to reduce simultaneity issues, introduce a wide set of firm-level and macroeconomic control variables, and include fixed effects to capture unobservable time-invariant firm/industry characteristics. Furthermore, we apply the dynamic system GMM estimator, which employs internal instruments derived from lagged levels and differences of the regressors.

Despite these efforts, we acknowledge that endogeneity cannot be entirely ruled out. Therefore, as a further step to reinforce the credibility of our findings, we implement an instrumental variable strategy using a two-stage least squares (2SLS) estimation procedure. This method allows us to isolate the exogenous variation in bank competition by relying on instruments that are correlated with bank competition but plausibly exogenous to corporate maturity mismatch decisions. Following previous authors [6,44], we select an instrument based on the five-year lag of financial development, measured by the ratio of domestic credit to GDP. This variable captures historical developments in the financial system, which are expected to influence current levels of bank competition but are less likely to be directly related to firms’ current investment-financing structures. The relatively slow-moving nature of financial sector evolution provides a sound basis for using past financial development as a valid instrument.

Table 7 presents the results of the second-stage 2SLS regressions. The significant coefficients on all three bank competition measures remain unchanged in sign, indicating that the main results hold when accounting for potential endogeneity through an instrumented framework. This supplementary analysis provides additional support for the robustness of our conclusions. The relationship between bank competition and corporate investment-financing maturity mismatch persists even after controlling for possible endogeneity, thereby reinforcing confidence in the consistency of our empirical findings.

**Table 7:** Robustness tests: instrumental variables.

	(1) IFMM	(2) IFMM	(3) IFMM
CR	−0.116***		
	(0.033)		
HHI		−0.436***	
		(0.124)	
Lerner			−0.282***
			(0.099)
Size	0.080***	0.078***	−0.032
	(0.006)	(0.007)	(0.042)
Leverage	−0.124***	−0.119***	0.162
	(0.028)	(0.028)	(0.111)
StateOwn	0.027	0.028	0.037
	(0.022)	(0.022)	(0.027)
ROA	−0.529***	−0.526***	−0.326***
	(0.059)	(0.059)	(0.106)
CashRatio	0.084**	0.087**	0.241***
	(0.036)	(0.036)	(0.071)
PPE	0.231***	0.234***	0.338***
	(0.030)	(0.030)	(0.052)
TobinQ	−0.028***	−0.028***	−0.024***
	(0.004)	(0.004)	(0.005)
Crisis	−0.122***	−0.150***	−0.159***
	(0.027)	(0.035)	(0.046)
Covid	0.031***	0.027***	−0.183***
	(0.010)	(0.010)	(0.069)
MoneySupply	0.053	0.090	2.577***
	(0.068)	(0.066)	(0.869)
StockReturn	0.025**	0.032**	0.123***
	(0.012)	(0.014)	(0.047)
Firm FE	Yes	Yes	Yes
Observations	6,377	6,377	6,377
Anderson canon. corr. LM statistic	1748.66***	1773.80***	25.38***
Cragg-Donald Wald F statistic	2483.37	2534.49	25.44

The dependent variable in all specifications is investment-financing maturity mismatch (IFMM). Significance levels: * p < 0.1; ** p < 0.05; *** p < 0.01.

## 5. Further analyses

### 5.1. Mechanism tests

In this section, we examine the underlying mechanism through which bank competition influences firms’ investment-financing maturity mismatch by focusing on corporate debt maturity structure. We predict that bank competition may increase mismatch by encouraging firms to rely more on short-term debt. To empirically test whether debt maturity mediates the relationship between bank competition and maturity mismatch, we conduct mediation analysis following the framework of Baron and Kenny [49]. This approach involves estimating a sequence of regressions to establish whether debt maturity transmits the effect of bank competition on mismatch. In the first step, we regress the mediating variable on competition measures to determine whether banking market structures significantly influence firms’ debt maturity profiles. The second step evaluates whether debt maturity itself has a direct effect on investment-financing maturity mismatch.

Table 8 presents the first set of mechanism tests, where the mediating variable is defined as the ratio of short-term debt to total debt [50]. This indicator captures the share of short-term borrowing in a firm’s total debt structure. The results in columns (1), (3), and (5) show that bank competition significantly increases the proportion of short-term loans, as reflected by the negative and statistically significant coefficients on CR, HHI, and Lerner. In the second-stage regressions, shown in columns (2), (4), and (6), the short-term debt ratio is positively associated with maturity mismatch, confirming that greater reliance on short-term borrowing contributes to higher mismatch. These findings support the notion that debt maturity acts as a transmission channel linking competition and mismatch.

**Table 8:** Mechanism tests: short-term debt maturity.

	(1)	(2)	(3)	(4)	(5)	(6)
	ShortDebtRatio	IFMM	ShortDebtRatio	IFMM	ShortDebtRatio	IFMM
Lagged DV	0.651***	0.158***	0.653***	0.158***	0.651***	0.160***
	(0.020)	(0.014)	(0.020)	(0.014)	(0.019)	(0.014)
ShortDebtRatio		0.186***		0.185***		0.188***
		(0.015)		(0.015)		(0.015)
CR	−0.395***	−0.379***				
	(0.108)	(0.146)				
HHI			−1.072***	−1.339**		
			(0.391)	(0.526)		
Lerner					−0.080***	−0.121***
					(0.022)	(0.033)
Firm-level controls	Yes	Yes	Yes	Yes	Yes	Yes
Country-level controls	Yes	Yes	Yes	Yes	Yes	Yes
Firm FE	Yes	Yes	Yes	Yes	Yes	Yes
Observations	6,380	6,380	6,380	6,380	6,380	6,380
Number of firms	498	498	498	498	498	498
Number of instruments	125	126	125	126	125	126
AR(1) test	0.000	0.000	0.000	0.000	0.000	0.000
AR(2) test	0.351	0.823	0.351	0.820	0.368	0.810
Hansen test	0.120	0.342	0.172	0.332	0.183	0.494

The dependent variables are short-term debt maturity (ShortDebtRatio) and investment-financing maturity mismatch (IFMM). Significance levels: ** p < 0.05; *** p < 0.01. The numbers in parentheses are standard errors clustered at the firm level.

In addition to the short-term debt share, we also explore whether the scale of short-term borrowing serves a similar mediating role. To this end, we use the natural logarithm of short-term debt as an alternative mediator. Table 9 reports the corresponding results. The estimates in columns (1), (3), and (5) reveal that bank competition leads to an increase in the absolute amount of short-term loans, with all three competition measures showing negative effects that are statistically significant. The second-stage regressions in columns (2), (4), and (6) indicate that the scale of short-term debt is positively related to maturity mismatch. These findings offer further support for the proposed mechanism: in more competitive banking markets, firms increase their short-term borrowing and rely more heavily on it to finance longer-term investments, thereby intensifying the maturity mismatch.

**Table 9:** Mechanism tests: short-term debt size.

	(1)	(2)	(3)	(4)	(5)	(6)
	LnShortDebt	IFMM	LnShortDebt	IFMM	LnShortDebt	IFMM
Lagged DV	0.475***	0.185***	0.474***	0.185***	0.476***	0.192***
	(0.020)	(0.017)	(0.020)	(0.017)	(0.020)	(0.014)
LnShortDebt		0.014***		0.014***		0.005***
		(0.003)		(0.003)		(0.001)
CR	−0.748***	−0.503***				
	(0.288)	(0.170)				
HHI			−2.960**	−1.630**		
			(1.216)	(0.634)		
Lerner					−2.002**	−0.071**
					(0.785)	(0.032)
Firm-level controls	Yes	Yes	Yes	Yes	Yes	Yes
Country-level controls	Yes	Yes	Yes	Yes	Yes	Yes
Firm FE	Yes	Yes	Yes	Yes	Yes	Yes
Observations	6,380	6,380	6,380	6,380	6,380	6,380
Number of firms	498	498	498	498	498	498
Number of instruments	125	126	125	126	125	126
AR(1) test	0.000	0.000	0.000	0.000	0.000	0.000
AR(2) test	0.125	0.857	0.125	0.841	0.127	0.705
Hansen test	0.358	0.251	0.349	0.283	0.218	0.461

The dependent variables are short-term debt size (LnShortDebt) and investment-financing maturity mismatch (IFMM). Significance levels: ** p < 0.05; *** p < 0.01. The numbers in parentheses are standard errors clustered at the firm level.

Overall, our mechanism tests support the view that bank competition increases corporate maturity mismatch by encouraging greater reliance on short-term debt. This finding strengthens our main results by identifying a clear transmission channel through which competitive banking environments influence firms’ debt structures. This mechanism aligns with theories of information asymmetry and relationship lending. In concentrated banking systems, lenders are more likely to form long-term relationships and extend long-term credit, especially when soft information is needed to assess risk [7,33]. By contrast, in competitive markets, banks often adopt transactional lending models, favoring short-term loans that require less information. Empirical evidence also supports this view. Petersen and Rajan [8] and Berlin and Mester [34] find that firms in competitive banking environments face tighter financing constraints. As competition rises, banks have fewer incentives to offer long-term credit, pushing firms toward short-term borrowing. This shift increases the likelihood of maturity mismatch by misaligning the duration of investments and liabilities.

Our results also help clarify the contrast with Li et al. [32], who find that bank competition reduces firms’ maturity mismatch by improving access to long-term loans. Their findings reflect the context of SMEs, which typically rely on relationship lending and benefit from stronger ties with banks under competitive conditions. In contrast, our analysis focuses on listed firms, which are more likely to access credit through transactional lending. SMEs often lack transparency and do not regularly disclose detailed financial information, requiring banks to depend on soft information obtained through repeated interactions. This relationship-based approach is essential for assessing creditworthiness in the absence of verifiable data [51]. Listed firms, however, usually provide audited financial statements and meet higher disclosure standards, allowing banks to rely on hard information and standardized metrics to make lending decisions [52]. As a result, banks are more likely to apply transactional lending models to listed firms, which are less dependent on long-term relationships with lenders. This distinction in lending practices contributes to the differing impacts of bank competition observed across firm types.

### 5.2. Heterogeneity tests

#### 5.2.1. Moderating effect of bank debt levels.

Understanding whether the relationship between bank competition and investment-financing maturity mismatch varies across firm characteristics is essential for refining our analysis. Heterogeneity tests help identify which firms are more susceptible to shifts in banking competition. Given the diversity in firms’ financial structures and access to credit, the influence of bank competition is unlikely to be uniform.

As the first step, we examine whether the share of bank debt moderates the effect of bank competition on maturity mismatch. We predict that firms with a larger proportion of bank debt are likely to be more directly affected by changes in banking market dynamics. As competition among banks intensifies, lending typically becomes more affordable, more accessible, and predominantly short-term [33]. Firms heavily reliant on bank financing are thus more inclined to adjust their borrowing behavior in response to these changes, unlike firms that primarily use internal funds or alternative market-based financing [53]. A common response among these firms is to replace long-term borrowing with cheaper short-term credit, thereby intensifying their maturity mismatch. While firms that rely on capital markets may maintain stability in their financing horizon, bank-dependent firms exhibit less flexibility and greater vulnerability to credit cycles and lending strategies driven by competition.

We empirically test this hypothesis by interacting bank competition variables with the bank debt ratio, defined as the share of bank debt in total assets. The results, presented in Table 10, align with our expectations. The interaction terms are statistically significant, and their coefficients mirror the direct effects of competition. Thus, these findings confirm that the maturity-mismatch impact of bank competition is more pronounced for firms with greater exposure to bank debt.

**Table 10:** Moderating effect of bank debt levels.

	(1) IFMM	(2) IFMM	(3) IFMM
Lagged DV	0.201***	0.201***	0.208***
	(0.015)	(0.015)	(0.015)
CR	−0.650***		
	(0.123)		
CR*BankDebt	−0.436***		
	(0.046)		
HHI		−2.312***	
		(0.515)	
HHI*BankDebt		−2.245***	
		(0.233)	
Lerner			−0.061*
			(0.037)
Lerner*BankDebt			−0.540***
			(0.053)
BankDebt	0.103***	0.105***	0.087***
	(0.016)	(0.016)	(0.015)
Firm-level controls	Yes	Yes	Yes
Country-level controls	Yes	Yes	Yes
Firm FE	Yes	Yes	Yes
Observations	6,380	6,380	6,380
Number of firms	498	498	498
Number of instruments	127	127	127
AR(1) test	0.000	0.000	0.000
AR(2) test	0.655	0.629	0.650
Hansen test	0.136	0.195	0.127

The dependent variable in all specifications is investment-financing maturity mismatch (IFMM). Significance levels: * p < 0.1; *** p < 0.01. The numbers in parentheses are standard errors clustered at the firm level.

#### 5.2.2. Moderating effect of financing constraints.

We next examine whether financial constraints moderate the relationship between bank competition and investment-financing maturity mismatch. Firms facing tighter financial constraints often struggle to secure long-term funding, making them more dependent on short-term borrowing [36]. When bank competition intensifies, lending conditions for short-term credit typically become more favorable. Financially constrained firms, with limited access to capital markets or long-term loans, are more inclined to rely on these short-term options to meet funding needs. This behavior can exacerbate the mismatch between their investment horizons and financing structures.

To capture financial constraints, we employ the KZ index and the SA index, as proposed by Kaplan and Zingales [54] and Hadlock and Pierce [55], respectively. Table 11 reports the estimation results. The interaction terms between the bank competition measures and both constraint indices are negative and statistically significant, confirming that the mismatch effect of bank competition is stronger among financially constrained firms. These findings are consistent with our earlier arguments and complement previous results concerning firms with high levels of bank debt. Collectively, the evidence reinforces the notion that financially vulnerable firms are particularly exposed to the maturity mismatch pressures induced by increased bank competition.

**Table 11:** Moderating effect of financing constraints.

	(1) CR	(2) HHI	(3) Lerner	(4) CR	(5) HHI	(6) Lerner
Lagged DV	0.188***	0.187***	0.196***	0.191***	0.196***	0.195***
	(0.014)	(0.014)	(0.014)	(0.013)	(0.013)	(0.013)
Competition	−0.637***	−2.506***	−0.024	−1.655***	−7.354***	−0.126***
	(0.114)	(0.486)	(0.034)	(0.136)	(0.600)	(0.045)
Competition*KZconstraint	−0.099***	−0.418***	−0.129***			
	(0.024)	(0.117)	(0.026)			
Competition*SAconstraint				−0.578***	−2.400***	−0.045***
				(0.059)	(0.245)	(0.014)
KZconstraint	0.064***	0.062***	0.072***			
	(0.010)	(0.010)	(0.009)			
SAconstraint				0.298***	0.267***	0.070***
				(0.031)	(0.028)	(0.018)
Firm-level controls	Yes	Yes	Yes	Yes	Yes	Yes
Country-level controls	Yes	Yes	Yes	Yes	Yes	Yes
Firm FE	Yes	Yes	Yes	Yes	Yes	Yes
Observations	6,380	6,380	6,380	6,380	6,380	6,380
Number of firms	498	498	498	498	498	498
Number of instruments	127	127	127	127	127	127
AR(1) test	0.000	0.000	0.000	0.000	0.000	0.000
AR(2) test	0.435	0.432	0.341	0.625	0.472	0.659
Hansen test	0.134	0.142	0.320	0.140	0.136	0.307

The dependent variable in all specifications is investment-financing maturity mismatch (IFMM). Competition measures are indicated at the top of the columns. Significance levels: *** p < 0.01. The numbers in parentheses are standard errors clustered at the firm level.

#### 5.2.3. Moderating effect of borrowing costs.

We further examine whether the effect of bank competition on maturity mismatch is moderated by firms’ borrowing costs. Firms that face higher borrowing costs are generally more financially constrained and more sensitive to fluctuations in credit conditions [56]. During periods of intensified bank competition, these firms are particularly responsive to reductions in lending costs and may be more inclined to adjust their capital structure by increasing reliance on short-term loans. The appeal of short-term credit lies in its typically lower interest rates under competitive lending conditions. Consequently, high-cost borrowers may exhibit a stronger tendency to shorten debt maturities in order to minimize financing expenses, which in turn can exacerbate their investment-financing maturity mismatch.

This behavioral response is consistent with previous research, which highlights that firms burdened by high financing costs have stronger incentives to reduce borrowing expenses and are more likely to substitute long-term borrowing with short-term alternatives when such options become available [35]. To quantify borrowing costs, we calculate the ratio of interest expenses to total debt. This firm-level indicator captures the average cost of borrowing and allows us to differentiate between high- and low-cost borrowers in our sample.

The empirical results, presented in Table 12, support this hypothesis. The interaction terms between the borrowing cost indicator and the bank competition measures are negative and statistically significant across specifications. These findings indicate that the mismatch-inducing effect of bank competition is stronger for firms with higher borrowing costs. In line with earlier results on bank debt exposure and financial constraints, this evidence further underscores the heterogeneity in the competition-mismatch relationship and highlights the particular vulnerability of financially disadvantaged firms to shifts in credit market dynamics.

**Table 12:** Moderating effect of borrowing costs.

	(1) IFMM	(2) IFMM	(3) IFMM
Lagged DV	0.178***	0.179***	0.181***
	(0.014)	(0.014)	(0.014)
CR	−0.202**		
	(0.093)		
CR*DebtCost	−0.041***		
	(0.013)		
HHI		−2.248***	
		(0.501)	
HHI*DebtCost		−0.024*	
		(0.014)	
Lerner			−0.102**
			(0.040)
Lerner*DebtCost			−0.051***
			(0.013)
DebtCost	0.068	−0.484	−0.041
	(0.494)	(0.401)	(0.112)
Firm-level controls	Yes	Yes	Yes
Country-level controls	Yes	Yes	Yes
Firm FE	Yes	Yes	Yes
Observations	6,380	6,380	6,380
Number of firms	498	498	498
Number of instruments	127	127	127
AR(1) test	0.000	0.000	0.000
AR(2) test	0.800	0.747	0.762
Hansen test	0.206	0.261	0.440

The dependent variable in all specifications is investment-financing maturity mismatch (IFMM). Significance levels: * p < 0.1; ** p < 0.05; *** p < 0.01. The numbers in parentheses are standard errors clustered at the firm level.

#### 5.2.4. Moderating effect of capital-intensive industries.

We extend our heterogeneity analysis by examining differences across industries in terms of capital intensity. Firms operating in capital-intensive sectors typically hold long-lived physical assets and thus require stable, long-term financing to support investment needs [57]. Because these firms face substantial risks when financing long-term projects with short-term debt, they are less likely to shift toward shorter maturities [48], even when bank competition makes short-term credit more accessible and affordable. As a result, the mismatch-inducing effect of bank competition is expected to be weaker in capital-intensive industries, where firms are more cautious about increasing reliance on short-term borrowing due to elevated refinancing risks associated with their asset structure.

To test this hypothesis, we classify industries by capital intensity using two firm-level indicators: the ratio of fixed assets to total assets, denoted as CapIntensive1 [58–60] and the ratio of fixed assets to sales, denoted as CapIntensive2 [61,62]. For each industry, we calculate the average of these ratios over the full sample period (2008–2024). Industries with average capital intensity above the median are classified as capital-intensive, while those below the median are treated as non-capital-intensive, following Liu et al. [63].

Table 13 reports the results. The interaction terms between the bank competition measures and the capital-intensive industry dummies are positive and statistically significant. These results confirm that the effect of bank competition on maturity mismatch is less pronounced in capital-intensive industries. Firms in these sectors appear more conservative in managing their financing horizons due to the longer duration of their investments. This finding suggests that they exhibit greater financial discipline in maintaining matched financing structures and are less likely to alter their maturity profiles in response to changing credit market conditions. These results reinforce the role of industry-specific characteristics in shaping how corporate strategies respond to banking competition.

**Table 13:** Moderating effect of capital-intensive industries.

	(1) CR	(2) HHI	(3) Lerner	(4) CR	(5) HHI	(6) Lerner
Lagged DV	0.181***	0.180***	0.178***	0.181***	0.180***	0.186***
	(0.014)	(0.014)	(0.014)	(0.014)	(0.014)	(0.014)
Competition	−0.305**	−1.021**	−0.381***	−0.313**	−1.127**	−0.428***
	(0.131)	(0.484)	(0.077)	(0.131)	(0.487)	(0.079)
Competition*CapIntensive1	0.138***	0.696***	0.107**			
	(0.025)	(0.128)	(0.043)			
Competition*CapIntensive2				0.120***	0.628***	0.103**
				(0.026)	(0.130)	(0.041)
CapIntensive1	0.032***	0.032***	0.102***			
	(0.009)	(0.009)	(0.026)			
CapIntensive2				0.020**	0.020**	0.114***
				(0.009)	(0.009)	(0.024)
Firm-level controls	Yes	Yes	Yes	Yes	Yes	Yes
Country-level controls	Yes	Yes	Yes	Yes	Yes	Yes
Firm FE	Yes	Yes	Yes	Yes	Yes	Yes
Observations	6,380	6,380	6,380	6,380	6,380	6,380
Number of firms	498	498	498	498	498	498
Number of instruments	127	127	127	127	127	127
AR(1) test	0.000	0.000	0.000	0.000	0.000	0.000
AR(2) test	0.661	0.661	0.764	0.788	0.790	0.737
Hansen test	0.343	0.354	0.299	0.348	0.375	0.544

The dependent variable in all specifications is investment-financing maturity mismatch (IFMM). Competition measures are indicated at the top of the columns. Significance levels: ** p < 0.05; *** p < 0.01. The numbers in parentheses are standard errors clustered at the firm level.

## 6. Conclusion

This study examines how bank competition influences corporate investment-financing maturity mismatch. Using panel data from 498 listed firms in Vietnam over the period 2008–2024, our findings consistently reveal that greater bank competition is associated with higher maturity mismatch. This core result holds under a wide array of robustness checks. We confirm its validity using alternative measures of the key variables, varying fixed-effects specifications, excluding crisis years, and adopting the instrumental variable approach. These tests affirm the reliability of the observed relationship between banking market structure and corporate investment-financing behavior.

Mechanism analysis demonstrates that bank competition influences firms’ liability structures by encouraging a shift toward short-term debt. In highly competitive banking environments, banks have less incentive to build long-term relationships and instead favor transactional lending, which emphasizes short-term, low-commitment loans. As long-term financing requires more information and monitoring, firms in such markets face greater difficulty obtaining long-term debt. Heterogeneity analysis reveals that this effect is stronger for firms with greater reliance on bank debt, those facing financial constraints, and those incurring high borrowing costs. Besides, the mismatch effect is weaker among firms in capital-intensive industries.

The findings of this study carry important implications for financial regulation, banking practices, and corporate financial management. While bank competition is generally associated with improved access to finance and greater efficiency, our results suggest a more nuanced effect: intensified competition may also encourage firms to rely more heavily on short-term debt, thereby increasing investment-financing maturity mismatch and exposing them to refinancing risks.

From a regulatory perspective, this highlights a potential trade-off between promoting competition and maintaining financial stability. Rather than focusing solely on enhancing market competition, policymakers may need to pay closer attention to how competition shapes the structure of credit, particularly the balance between short-term and long-term lending. Strengthening macroprudential oversight and fostering the development of long-term credit markets could help mitigate the unintended consequences of excessive reliance on short-term financing.

At the same time, the results point to important considerations for banks. Competitive pressures may push banks toward short-term, transaction-oriented lending strategies, which are easier to price and manage in the short run. However, such strategies can weaken long-term lending relationships and increase borrowers’ exposure to liquidity risks. Expanding long-term loan offerings, improving risk assessment for long-term projects, and maintaining stable lending relationships could help banks better support firms’ investment needs while reducing systemic vulnerabilities.

For firms, especially those that are financially constrained or heavily dependent on bank financing, the tendency to substitute long-term debt with short-term borrowing can increase exposure to liquidity shocks. In such environments, more proactive debt maturity management becomes essential. Diversifying funding sources, extending debt maturities when feasible, and avoiding excessive reliance on short-term financing for long-term investments can help firms strengthen their financial resilience.

More broadly, the prevalence of maturity mismatch reflects a structural limitation in many emerging markets, namely the insufficient availability of long-term financing. Addressing this issue requires coordinated efforts to deepen financial markets and enhance institutions that support long-term credit provision. Improving access to long-term funding would enable firms to better align financing structures with investment horizons, thereby reducing mismatch risks and supporting more sustainable economic growth.

A key limitation of this study is the absence of loan-level data, which restricts our ability to directly observe the specific maturity terms of individual borrowings. Although we analyze the overall scale and proportion of short-term debt, more granular data would enable a more detailed examination of how bank competition affects the structure and duration of corporate debt. Addressing this limitation in future research would provide deeper insights into the mechanisms through which bank competition shapes corporate financing strategies.
